# Impact of an enhanced sobriety checkpoints programme and publicity campaign on motor vehicle collisions, injuries and deaths in Leon, MX: a synthetic control study

**DOI:** 10.1136/ip-2023-045019

**Published:** 2024-07-22

**Authors:** D. Alex Quistberg, Carolina Perez-Ferrer, Usama Bilal, Jordan Levi Rodriguez Hernandez, Yenisei Ramírez-Toscano, Luz Mery Cardenas Cardenas, Isabel Junquera-Badilla, Goro Yamada, Tonatiuh Barrientos-Gutierrez, Ana V Diez Roux

**Affiliations:** 1 Urban Health Collaborative, Dornsife School of Public Health, Drexel University, Philadelphia, Pennsylvania, USA; 2 Environmental & Occupational Health, Dornsife School of Public Health, drexel University, Philadelphia, Pennsylvania, USA; 3 Centro de Investigación en Salud Poblacional, Instituto Nacional de Salud Publica, Cuernavaca, Morelos, Mexico; 4 Department of Epidemiology & Biostatistics, Dornsife School of Public Health, Drexel University, Philadelphia, Pennsylvania, USA; 5 Consejo Nacional de Ciencia y Tecnología, Instituto Nacional de Salud Publica, Mexico City, Distrito Federal, Mexico

## Abstract

**Objective:**

Drunk driving is a major cause of road traffic injuries and deaths in Latin America. We evaluated the impact of a drunk driving intervention in Leon, Mexico on road traffic safety.

**Methods:**

The intervention included increased drunk driving penalties, enhanced sobriety checkpoints and a young adult-focused mass media campaign, beginning 19 December 2018. We created a synthetic control Leon from 12 Mexican municipalities from a pool of 87 based on similarity to Leon using key predictors from 2015 to 2019. We assessed the effect of the intervention on road traffic collisions overall and collisions with injuries, deaths and involving alcohol, using data from police, insurance claims and vital registration.

**Results:**

As compared with the synthetic control, Leon experienced significant postintervention lower police-reported total collision rate (17%) and injury collisions (33%). Alcohol-involved collisions were 38% lower than the synthetic control. Fatal collisions reported by police were 28% lower while vital registration road traffic deaths were 12% lower, though these declines were not statistically significant. We found no impact on insurance collision claims. There was heterogeneity in these changes over the evaluation year, with stronger initial effects and weaker effects by the end of the year.

**Conclusions:**

Drunk driving policies in Leon led to fewer traffic collisions and injuries during the first year of implementation, with a weakening of this effect over time, similar to interventions in high-income settings and other Latin American countries. Supporting the expansion of similar policies to other cities in the region could improve road safety.

## Introduction

Road traffic injuries are a major cause of disability and death in Latin America and the Caribbean (LAC).^1–4^ Alcohol is involved in up to 50% of road traffic incidents in the region, with some studies finding nearly 60% of drivers with detectable alcohol in their blood and 27% of disability-adjusted life-years due to alcohol were road traffic injury related.^3–7^ Despite this, few studies have examined the effect of alcohol enforcement policies on road traffic safety in the region.^3 7–10^

Legal limits for blood alcohol content (BAC) accompanied by police enforcement (eg, sobriety checkpoints) are effective in reducing drunk driving.^2 11–17^ In LAC, few countries meet the WHO recommended 0.05 g/dL standard^2^ while others have the limit set at 0.08 g/dL.^2^ Sobriety checkpoints can reduce fatal collision rates by up to 71% depending on the intensity of enforcement and the time period examined.^13–16^ Sobriety checkpoints may also result in a 10%–15% reduction in all injury-causing collisions.^17^ Impacts can be observed within months after implementation if accompanied by sustained enforcement (eg, multiple, simultaneous, random checkpoints), though effects may taper off or be reversed if enforcement is not maintained.^11 13 18^

Sobriety checkpoint use in LAC has increased in the past decade, though there are few published evaluations.^6 9 10^ In Mexico, the federal government has provided guidelines for the enforcement of drunk driving policies (https://www.gob.mx/salud/acciones-y-programas/secre-tariado-tecnico-del-consejo-nacional-para-la-prevencion-de-accidentes-102486); however, laws and enforcement governing drunk driving are set locally. In the municipality of Leon, Guanajuato, some alcohol control policies have been implemented over the past decade and two evaluation studies found a slight decrease in total police-reported collision rates, but no impact on collisions involving a death or injury.^9 19^

**Figure 1:**
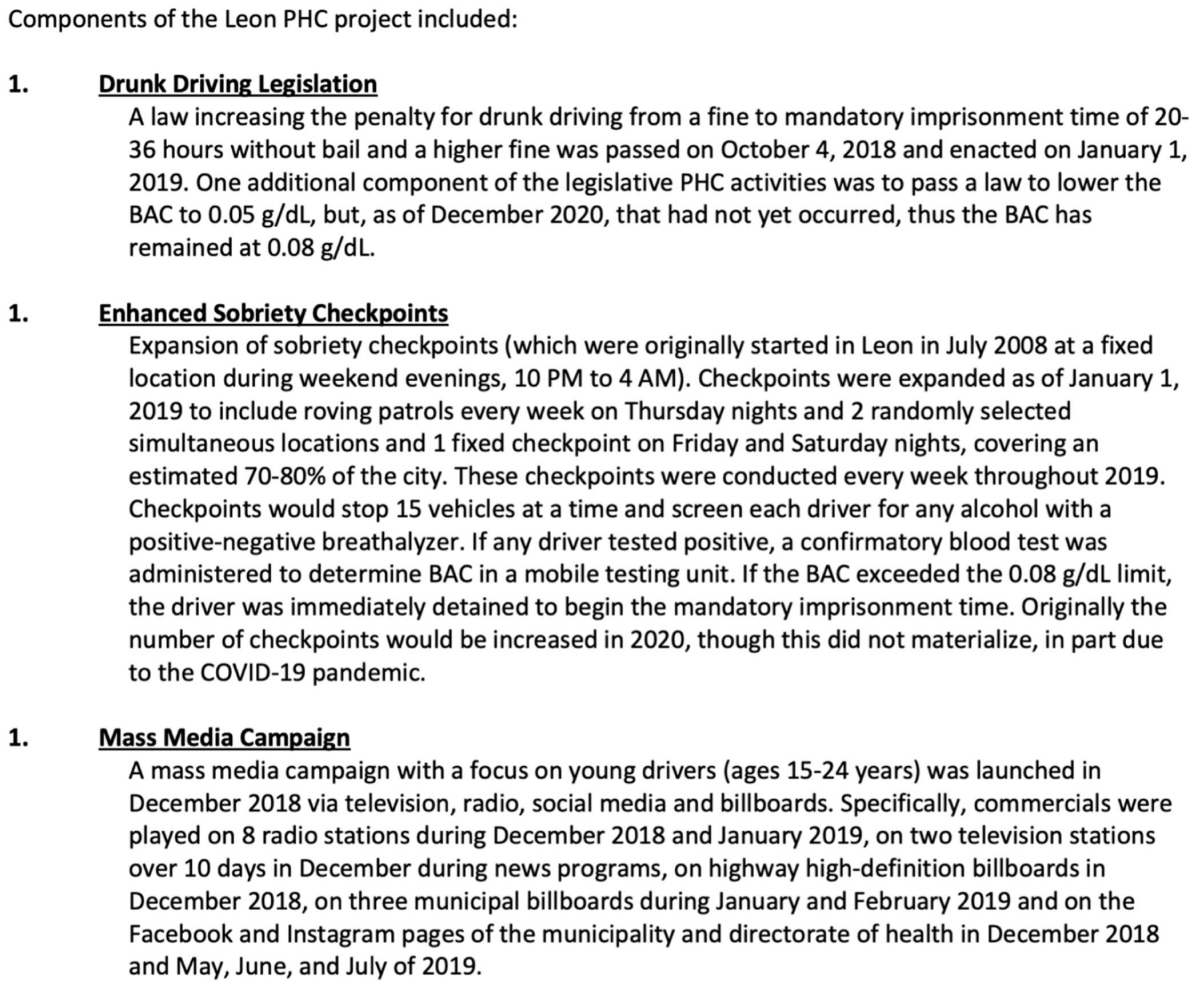
Description of the drunk driving intervention components implemented in Leon, Mexico, 2018–2019. BAC, blood alcohol content; PHC, Partnership for Healthy Cities.

In March 2018, Leon joined the network of the Partnership for Healthy Cities, a Bloomberg Philanthropies programme focused on implementing policies to prevent and reduce non-communicable diseases and injuries in cities with a focus on strengthening drunk driving laws (figure 1). We evaluate the impact of the programme (which included stricter enforcement, expanded check points and a mass media campaign) on road traffic collisions, collisions with injuries, fatal collisions and alcohol-involved collisions. Identifying the impact of policy changes in real-life settings is critical to efforts to inform implementation of similar policies in cities across the region.

## Methods

### Setting and intervention

Leon has nearly 1.6 million inhabitants and located in central Mexico. The study period is from 1 January 2015 to 31 December 2019, with the preintervention period occurring 1 January 2015–31 December 2018 and the postintervention period is from 1 January 2019 to 31 December 2019 (online supplemental appendix figure 1). The intervention included increased penalties for drunk driving (including mandatory imprisonment for at least 24 hours), the expansion of sobriety checkpoints in Leon and a limited mass media campaign (figure 1). Because the mass media campaign was limited and not sustained, our evaluation did not consider its effects.

### Study design

We used the synthetic control method (SCM) to evaluate the intervention by comparing the intervention unit (the municipality of Leon) to a control unit (‘a synthetic control’ henceforth referred to as ‘synthetic Leon’), that represents a counterfactual version of Leon, providing an estimate of the levels of the outcomes that would have been observed in Leon had the intervention not occurred.^20 21^ The main idea of the SCM is to construct a synthetic control, which is a weighted combination of the control units, that resembles the intervention unit in relevant preintervention characteristics.^20 21^ This allows us to estimate the causal effect of the intervention by evaluating the outcomes during the post-treatment period. This method has been used to estimate the causal effect of gun control laws on US firearm deaths and avoids issues with parallel trends assumption in some quasi-experimental designs and does not require a large sample size.^20–24^ Synthetic Leon was created by combining information from predictors of a donor pool of control units that are similar to the intervention unit to create weights for the control units.^21 22 25^ The donor pool consisted of the core municipality of 91 urban areas with ≥100 000 inhabitants in Mexico previously identified for the SALURBAL study (Salud Urbana en America Latina, Urban Health in Latin America).^26^ A core municipality of an urban area was defined as the municipality for which the urban area was named for, the municipality with the largest population of the urban area, or the municipality from which the urban area originates. Mexico City’s urban area was excluded due to population size. Urban areas in the state where Leon is located (Guanajuato State) were excluded (N=3) to avoid impact of any spill-over effects. The remaining 87 core municipalities were narrowed to 13 that were most like Leon with respect to key predictors (see below and online supplemental appendix table 1). To be included as a control unit, the core municipalities had to have values within 1 SD of the value of Leon on predictors that were selected based on existing evidence and relationships with the road traffic collision outcomes (online supplemental appendix figure 2). One of the 13 municipalities was excluded due to a period of severe violence during the study period, potentially impacting police reporting. The final donor pool of municipalities consisted of 12 municipalities and final synthetic control weights for each municipality are in online supplemental appendix table 2.

### Data sources and description

#### Outcomes

Primary outcomes were (1) monthly rate of automobile collision insurance claims calculated as the total number of collisions per 100 000 registered vehicles covered by the insurance company; (2) monthly rate of police-reported collisions per 100 000 registered motor vehicles and (3) bimonthly rate of road traffic deaths per 100 000 population from vital registration. Secondary outcomes included monthly rate of collisions involving injuries in each of insurance and police data and monthly rate of collisions involving a death or alcohol from police reports.

### Police data

We created monthly rates of police-reported collisions, collisions involving injuries, and collisions involving deaths or alcohol, per 100 000 registered motor vehicles. Police reports collected by the Instituto Nacional de Estadística y Geografía (INEGI, Mexico’s national statistical agency, https://www.inegi.org.mx/programas/accidentes/) were obtained and included for each reported collision the number of people involved, the number of vehicles involved by type, the number of injuries and deaths of driver, passenger, pedestrian or cyclist and whether alcohol was involved. Typically, police in Mexico only respond to collisions that involve a death at the scene, severe injuries requiring medical care or that require a tow-away of a damaged vehicle, though this varies by municipality.^27^ Annual vehicle registration data were obtained from INEGI (https://www.inegi.org.mx/programas/vehiculosmotor/) and monthly counts for each municipality were created via interpolation.

### Insurance claims data

We created monthly rates of insurance collision claims and insurance collision claims involving injuries per 100 000 registered automobiles covered by insurance. Automobile collision insurance claims data were from AXA (https://i2ds.org/datos-abiertos/), an insurance company with national coverage. Insured drivers in collisions reported to insurance adjusters who submitted the claims, which included information about the number of vehicles and people involved, damages to the vehicle, injuries, hospitalisations, deaths and other information about the collision. Claims were geographically linked to municipalities using latitude and longitude from the record (92% of claims) or the municipality name and state name (8% of claims). We estimated the number of registered automobiles covered by AXA insurance by using data on motor vehicle registration at the municipality level obtained from INEGI, and on the proportion of vehicles covered by any automobile insurance at the state level (see online supplemental appendix page 8 for more details, https://centroestadisticoamis.mx/tablero-dinamico-reportes/).

### Death data from vital registration

We used mortality records from INEGI and population post-censal projections or estimates from the Consejo Nacional de Población^26^ to calculate bimonthly road traffic deaths per 100 000 population among residents of Leon and the control municipalities. Road traffic death data used in the study have been described elsewhere^28^ and were identified using the International Classification of Diseases version 10 codes V01–V89 that cover land transport causes of death with some exceptions (online supplemental appendix page 8). Road traffic death data were corrected for undercounting using death distribution methods.^29–31^ Ill-defined external causes of death that could possibly include road traffic deaths (online supplemental appendix table 3) were multiply imputed 100 times by categories of age and sex. For analyses and for simplicity, we used the mean of these 100 imputations due to low variation between redistributions.

### Predictors

We developed a conceptual model to select key predictors to be considered for refining the donor control pool for synthetic Leon (online supplemental appendix figure 2). Urban form measures examined included urban patch density (fragmentation of the urban area), area-weighted mean nearest neighbour distance (isolation of urban patches from one another) and size of urban area. Population measures included urban population, proportion of population 15–34 years old and proportion of population male. Road network measures included intersection density, congestion index (average delay to travel between randomly selected points in the city during rush hour vs non-rush hour times) and street length (metres). Social environment measures from the 2010 census included the proportion of the population with at least a high school education. Alcohol outlet density is the only time-varying predictor (annual). These measures and their sources are described in more detail in prior SALURBAL publications and online supplemental appendix table 2.^26^ The final donor municipalities had to have values within one SD of Leon’s values across all predictors.

### Statistical analysis

As described earlier, the final donor pool to create the synthetic control included 12 municipalities. Two municipalities had no reported collisions during certain months of the study due to police strikes (one in January and February 2015, the other in December 2019). Dropping those municipalities from analyses resulted in similar trends as excluding those months from the analysis, though they did not match the preintervention trend as well (higher root mean square prediction error, RMSPE), thus the final study period in analyses was from March 2015 to November 2019. In addition to the key predictors, we also included the preintervention trend of the outcome as a predictor in our models, which is recommended for SCM analyses.^20 32^ Based on the performance of various parameterisations of the preintervention trend of the outcome (mean of incident rates of each preintervention year, 2015–2016, 2017–2018 and 2015–2018) using the RMSPE, we found that the mean incident rate for 2015–2016 was the best fitting preintervention predictor. We calculated the average monthly per cent difference between Leon and synthetic Leon for each outcome postintervention to compare trends.

We addressed known challenges to assess statistical significance in the SCM^21^ by implementing complementary approaches to evaluate the likelihood that any intervention effects we observed were due to chance.^33^ We conducted placebo tests,^20 21^ where each donor control municipality was treated as if it was the intervention unit, creating a synthetic control separately for each donor unit. If the gap observed when comparing Leon to synthetic Leon is larger than the gaps observed when comparing each of the donor municipalities to its own synthetic control, we concluded that it was unlikely that the effect observed in Leon was due to chance. In some instances, placebo runs for other control municipalities had a poor fit during the preintervention period (very large RMSPE), making it challenging to assess the postintervention gap. To interpret our placebo tests, we followed the recommendation from previous literature and excluded any control unit with a preintervention RMSPE more than five times the RMSPE error of Leon.^20^ This led to the exclusion of Torreón from the placebo tests for police-reported data of total collisions and collisions with injuries. However, for the remaining outcomes, the placebo tests included all control units. The large preintervention RMSPE for Torreón can be attributed to its high collision rates in police-reported collisions, which is considerably higher than most other control units (see online supplemental appendix figure 3). Consequently, no combination of municipalities in our sample was able to reproduce Torreón’s preintervention collision trends. We also examined the post-RMSPE/pre-RMSPE ratios by plotting the distributions for each placebo analysis. This approach offers the advantage of including all control units, irrespective of their preintervention RMSPE and eliminates the need to choose a cut-off for placebos with a poor fit. In a third approach recommended by Firpo and Possebom,^33^ we conducted a test of the null hypothesis that there is no intervention effect under the assumption that the effect of the intervention remains the same throughout the entire intervention evaluation period. The p value of the hypothesis test was calculated for each outcome and statistical significance was considered for p values <0.05.

We repeated all analyses using an interrupted time series (ITS) approach as a sensitivity analysis.^34^ We tested if the intervention resulted in either or both a level change and trend change (the trend is shifted lower and the trend decreases) compared with the preintervention period. We tested these hypotheses by examining trends in Leon. We used generalised linear models with a Poisson distribution and robust SEs, and count of registered vehicles as an offset and repeated analyses with seasonally adjusted counts using a Fourier transformation with a sine function.^34^ While we considered expanding this ITS analysis to include a control (an average of control units), preintervention trends were not linear, thus a comparison between trends of the control average and Leon was not possible.^35^

For an additional sensitivity analysis on the timing of the intervention, we created a synthetic control composed of all 87 donor pool municipalities. We also tested different intervention start dates (see online supplemental appendix page 11) according to when legislation was passed (1 October 2018) and when the mass media campaign began (19 December 2018).

### Patient and public involvement

No patients or the general public were involved in the study design or analyses. Research results will be disseminated to local government, news organisations and road safety advocacy groups.

## Results

Leon had similar predictor values compared with the synthetic control except for intersection density, population density and urban area (table 1).

The synthetic control units for each outcome better matched Leon’s values compared with a simple average (online supplemental appendix table 5). In general, there were higher frequencies of collision insurance claims (online supplemental appendix figure 4) than police-reported collisions across all municipalities (online supplemental appendix figure 3). In some municipalities, there were months with no observed claims of injuries. Leon typically had lower rates of road traffic deaths from vital registries per 100 000 population than control municipalities (online supplemental appendix figure 5).

Preintervention trends for insurance claim collisions (total and those reporting injuries) for Leon and synthetic Leon were similar (online supplemental appendix figure 6). There was a little change in trends in claims postintervention implementation (table 2). Placebo tests (online supplemental appendix figure 1) confirmed that there was no clear evidence of differences in insurance-reported collisions (total and injury), as did post-RMSPE/pre-RMSPE ratios.

**Figure 2:**
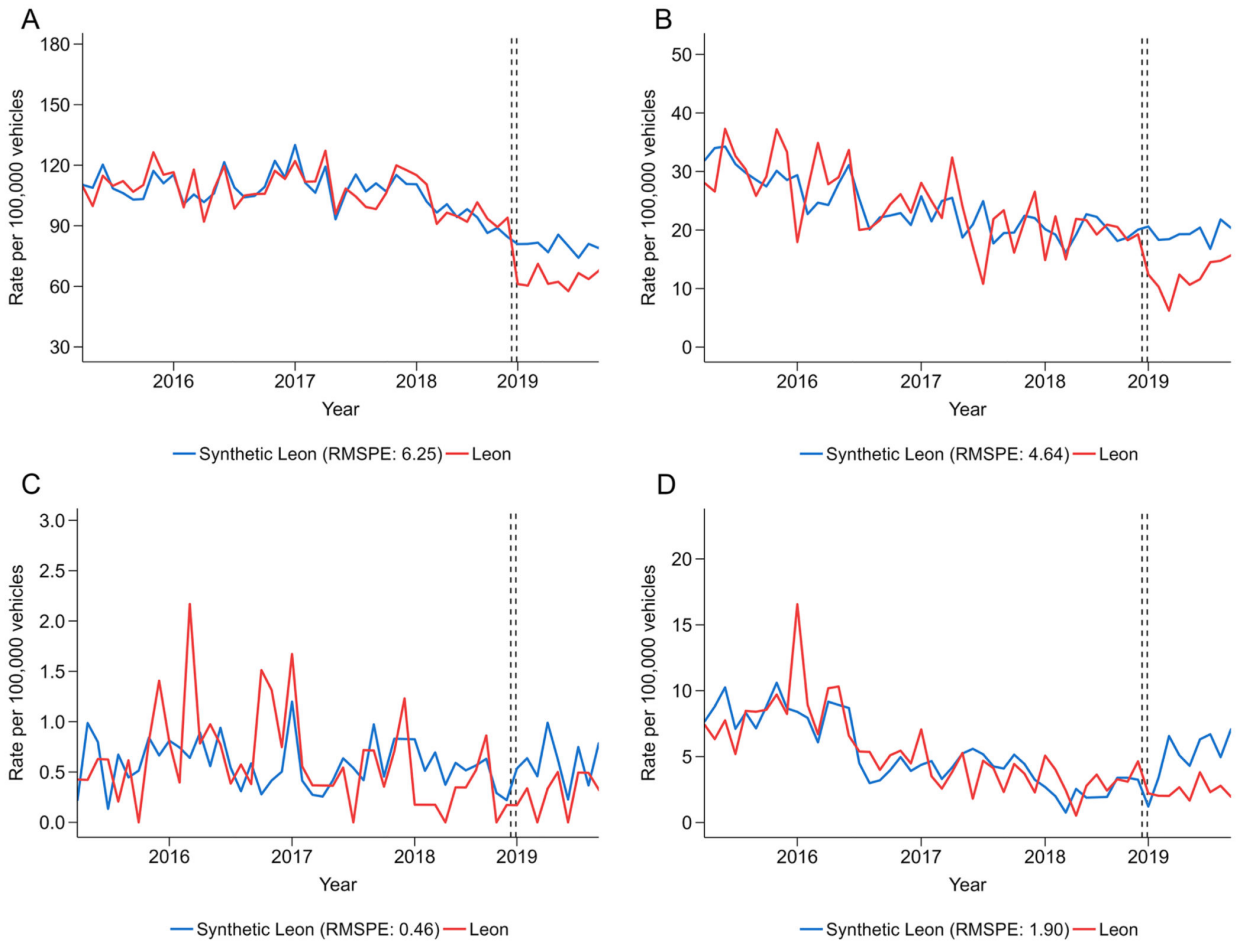
Monthly rates of police-reported collisions by (A) total, (B) injury, (C) fatal and (D) alcohol in Leon versus synthetic Leon, March 2015–November 2019, before and after the implementation of an intervention to reduce drunk driving in January 2019. Lower values indicate a closer match of synthetic Leon to Leon during the preintervention period. RMSPE, root mean square prediction error.

**Figure 3:**
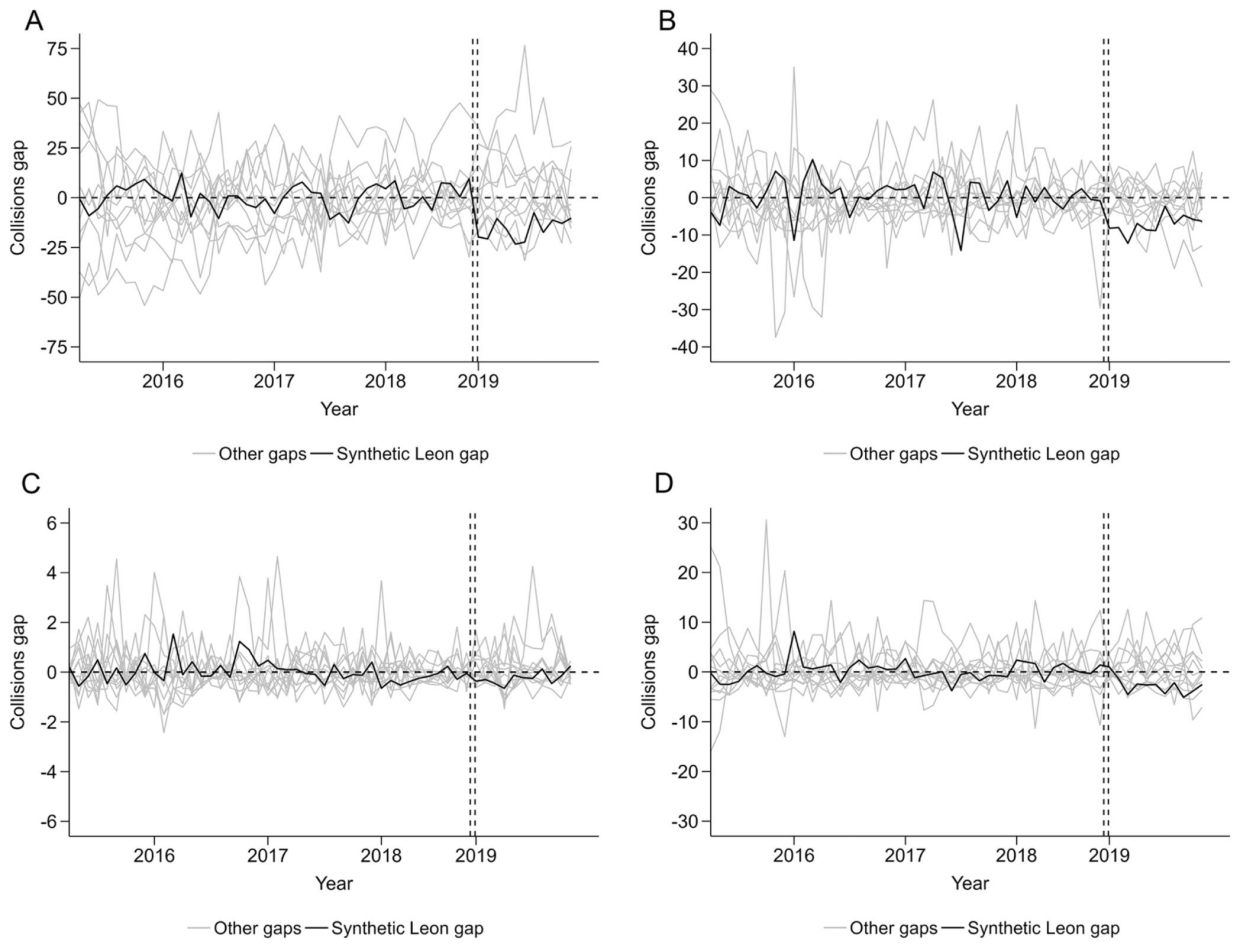
Placebo tests of police-reported collision rates, March 2015–January 2019 in Leon, Mexico and municipalities that contributed to the synthetic control. Each figure shows difference (gap) by (A) total (B) injury, (C) fatal and (D) alcohol collisions by month between Leon and synthetic Leon and between each control municipality and its own synthetic control (placebo tests, labelled ‘other gaps’). Gap is expressed as differences in rates per 100 000 insured vehicles. Note, all municipalities (12/12) had preintervention RMSPE <5 times the RMSPE of Leon. RMSPE, root mean square prediction error.

For police-reported collisions, preintervention trends were comparable between Leon and synthetic Leon (figure 2). Total police-reported collisions on average were 17% lower postintervention compared with synthetic Leon (table 2), though the monthly trend in Leon showed a sharp initial decrease and a later increase with monthly rates remaining lower than preintervention rates and synthetic Leon. Postintervention, collision rates in Leon resulting in injuries, involving alcohol and fatalities were 33%, 38% and 28% lower than synthetic Leon per month on average, respectively, though with similar initial declines and later increases as in total police-reported collisions. For alcohol-involved collisions, it should be noted that the difference between Leon and synthetic Leon in the postintervention trend was mostly due to an increase in alcohol-related collisions in synthetic Leon rather than a decrease in Leon. Placebo tests suggested that the differences between Leon and synthetic Leon were no clear evidence of differences in total collisions and injury collisions (figure 3), confirmed by post-RMSPE/pre-RMSPE ratios (online supplemental appendix figure 7) and the hypothesis tests suggested by Firpo and *et al*^33^ that showed statistically significant (p<0.05) intervention effects for total and injury collisions.

Similar to insurance claims and police reports, road traffic death trends in the pre-intervention period were similar between Leon and synthetic Leon (online supplemental appendix figure 8). Road traffic deaths from vital registries decreased in Leon by 11.6% compared with synthetic Leon in the postintervention period (table 2). Placebo tests suggested this decrease was not clearly different from donor municipalities, as did the post-RMSPE/pre-RMSPE ratio.

The ITS results (online supplemental appendix table 6), showed no significant postintervention differences in insurance data, nor alcohol-related or fatal collisions using police data. However, we observed a statistically significant postintervention reduction for total collisions and injury collisions using police data (online supplemental appendix figure 9), with an immediate decrease in collisions of 35% (IRR 0.65, 95% CI 0.57 to 0.73) and 44% (Incident Rate Ratio [IRR] 0.56, 95% CI 0.41 to 0.75), respectively. The trend after the intervention started, however, increased by 2% (IRR 1.02, 95% CI 1.00 to 1.04) and 9% (IRR 1.09, 95% CI 1.04 to 1.12) per month on average, respectively, for total and injury collisions. The rate of injury collisions exceeded preintervention rates by the end of the evaluation period. Fatal and alcohol collisions also had increasing trends later in the postintervention period. While the rate of road traffic deaths from vital registry decreased after the intervention was started (11.5%), this was not statistically significant nor was the trend. The synthetic Leon composed of all 87 donor pool municipalities performed very poorly compared with the synthetic Leon created for the main analyses from 12 municipalities with a much higher RMSPE. Varying the start dates of the intervention did not have a significant impact on the main study results (online supplemental appendix table 7). We have also included the results of the controlled ITS (online supplemental appendix figure 10).

## Discussion

The Leon drunk driving intervention resulted in significantly lower police-reported total collisions and injury collisions compared with a synthetic control, though the month-to-month trends suggest the intervention effect weakened by the end of the 12-month period as there were observed increases by the last few months compared with initial months. Differences in police-reported alcohol-related and fatal collisions were not significant and we observed no impact on insurance claim-reported collisions. Our sensitivity analysis using ITS supported these findings also suggested a weakening of the intervention effect by the end of the evaluation period as the average monthly trend increased postintervention. The discrepancy between police reports and insurance claims may be due to differences in the severity of collisions reported in each dataset. Prior literature suggests that all collisions can be reduced by at least 10% after implementing sobriety checks while impacts on more severe collisions can be much more substantial.^14 15 36^ Police-reported collisions tend to be more severe, thus the stronger effect is unsurprising.^37^ Pre-existing, limited sobriety checks in Leon may have already reduced less severe collisions that would be reported in claims. Sobriety checkpoints in prior studies have reduced all types of collisions, possibly because increased police presence affects driver behaviours, overall.^15^ Another possibility is that insurance claims originate from insurable vehicles driven by safer drivers than the average population of drivers. Only one-third of all vehicles are insured in Mexico and there is substantial local heterogeneity,^38^ thus the types of vehicles covered (in terms of their likelihood of collisions) could vary over time in a way that is aligned with the intervention. Insurance collision data may also capture more non-serious collisions or even over-report collisions (reported only for insurance reimbursement purposes) that are less likely to be impacted by the intervention.

There are important limitations to this study primarily related to the available outcome data. We could not test for long-term impacts due to COVID-19 pandemic, which resulted in a pause in sobriety checks and substantial changes in underlying traffic patterns compared with prior years.

Police-reported collisions may under-report even serious collisions due to other demands on police, resources to record and report collisions, and factors such as violence. If under-reporting does not differ systematically over time in a way aligned with the intervention, preintervention and postintervention comparisons using these data may still be valid. Police-reported alcohol use had substantial missing values, thus alcohol-involved collisions were likely undercounted, especially considering estimates that 20% of fatal collisions in Mexico involve alcohol.^39^ Another possible limitation is that our analytical approach missed the mass media effects by not considering them in analyses. However, our results were robust to modifying the definition of the preintervention and postintervention periods slightly to account for possible impacts of passing the legislation in October (even before it was implemented) or for early media campaign impacts in December 2018 and prior literature suggests mass media campaigns alone, particularly for such a brief time, do not have any impact on collisions.^40^ Furthermore, our dual identification strategy using SCM and ITS with analogous results lends robustness to these findings. ITS, as a method without controls, is not sensitive to the choice of controls and predictor variables while SCM is not as sensitive to model specification as ITS. Finally, vehicle miles travelled or trips taken would have been a better denominator for our analyses, but those data are not available in most municipalities in Mexico, thus, we relied on vehicle registration and insurance policies as proxies.

## Conclusions

A policy and enforcement intervention to reduce drunk driving implemented in the city of Leon, Mexico, had a substantial, immediate impact on severe collisions, though we could not evaluate long-term sustainability. Further complementary policies may be needed to reduce drunk driving, such as lowering the BAC, increasing the number of sobriety checkpoints and ensuring programme sustainability.^14 36 41^ Our findings suggest that cities can obtain additional road safety improvements by expanding existing sobriety checkpoint programmes.

## Supplementary Material

This content has been supplied by the author(s). It has not been vetted by BMJ Publishing Group Limited (BMJ) and may not have been peer-reviewed. Any opinions or recommendations discussed are solely those of the author(s) and are not endorsed by BMJ. BMJ disclaims all liability and responsibility arising from any reliance placed on the content. Where the content includes any translated material, BMJ does not warrant the accuracy and reliability of the translations (including but not limited to local regulations, clinical guidelines, terminology, drug names and drug dosages), and is not responsible for any error and/or omissions arising from translation and adaptation or otherwise.

Data Supplement 1

## Figures and Tables

**Table 1:** Predictor characteristics of Leon, synthetic control units by outcome

		Synthetic Leon (n=12)*								
		AXA insurance claims			Police-reported collisions					Vital registration
Predictor	Leon	Total	Injuries		Total	Injuries	Fatal	Alcohol		Deaths
Intersection density, 2018†	19.2	15.6	10.9		15.8	13.2	16.2	16.3		11.0
Population density, 2018†	8675	6898	6788		6670	7306	6592	6494		6659
Patch density, 2011 (fragmentation)	0.6	0.6	0.6		0.5	0.5	0.6	0.6		0.5
Alcohol outlets density, 2016/2018†	0.8	0.5	0.4		0.5	0.4	0.6	0.6		0.5
% population male, 2018	47.8	47.7	48.1		47.8	47.7	47.8	47.8		49.5
% population 15–34 years old, 2018	35.4	35.8	35.9		35.4	35.8	35.4	35.4		36.0
Total urban area, 2011 (km^2^)	16 504	9394	10 818		8733	9518	9131	9432		9561

Values for predictors vary between synthetic control units for each outcome due to differing weights of each donor municipality. See online supplemental appendix table 1 for more details about each predictor and online supplemental appendix table 2 for more details on the weights for each municipality within each synthetic control unit.

* Because the synthetic control set weights may be different for different outcomes, the characteristics of each synthetic control vary and thus are shown by outcome.

† Per km^2^

**Table 2:** Average percentage difference between Leon and synthetic Leon in the postintervention period

Insurance collision claims	
Total	+3.4%
Injuries	+3.6%
Police-reported collisions	
Total	−17.0%*
Injuries	−33.0%*
Fatal	−28.0%
Alcohol	−38.0%
Vital registration road traffic deaths	
Deaths	−11.6%

* A statistically significant difference between Leon and synthetic Leon in in null hypothesis test (p<0.05).

## Data Availability

Data are available on reasonable request. All data used in this study were from publicly available sources described in the manuscript. Analytical data created by the authors can be shared on request.

## What is already known an this topic

⟹ Drunk driving is involved in 20%–50% of collisions in Latin America and the Caribbean.
⟹ Policies setting blood alcohol content limits and legal consequences paired with sobriety checkpoints are effective at reducing drunk driving and reducing road traffic deaths.
⟹ Countries and cities in Latin America and the Caribbean are implementing or considering sobriety checkpoints to enforce blood alcohol content limits, but there is limited evidence of their effects.

## What this study adds

⟹ Stricter legal consequences and an expanded sobriety checkpoints programme led to a reduction in road traffic collisions and injuries over a 1-year postintervention implementation period.

## How this study might affect research, practice or policy

⟹ Cities with existing laws and checkpoints programmes may find further road safety improvements with stricter legal consequences and expanding the reach of current sobriety checkpoints programmes.
