# Supplementary material for: Impact of an enhanced sobriety checkpoints programme and publicity campaign on motor vehicle collisions, injuries and deaths in Leon, MX: a synthetic control study
Source: Inj Prev. Author manuscript; Available in PMC 2026 Feb 1. (PMC7617682; doi:10.1136/ip-2023-045019)
Supplement: Supplementary file 1 [file EMS204986-supplement-Data_Supplement_1.pdf]

Supplemental File for:

The impact of an enhanced sobriety checkpoints program and publicity campaign on motor vehicle collisions, injuries, and deaths in Leon, MX: A synthetic control study

Quistberg DA, Perez-Ferrer C, Bilal U, Rodriguez Hernandez JL, Ramirez Toscano Y, Cardenas Cardenas LM, Junquera Badilla I, Yamada G, Barrientos-Gutierrez T, Diez Roux AV

Supplemental File Contents

1) Appendix Figure 1. Leon intervention and evaluation timeline for main analyses

2) Appendix Table 1. Description of key predictors used to create synthetic Leon

3) Appendix Figure 2. Conceptual model of pre-intervention predictors, policy and road traffic collisions and deaths

4) Appendix Table 2. Weight assigned to synthetic control municipalities by outcome

5) Insurance Claims Denominator Estimations

6) Description of specific ICD-10 codes used to identity road traffic deaths

7) Appendix Table 3. Ill-defined ICD-10 death codes redistributed.

8) Appendix Figure 3. Time trends by municipality for police-reported collisions claims 2015-2019 by total and injury-causing collisions

9) Description of Varying Time Periods for Sensitivity Analyses

10) Appendix Table 4. Expanded version of Table 1 that includes Control Average results compared to synthetic Leon and Leon.

11) Appendix Figure 4. Time trends by municipality for police-reported collisions 2015-2019 by total, injury-causing, fatal, and alcohol-involved collisions, 2015-2019

12) Appendix Figure 5. Bi-Monthly rates of road traffic deaths from vital registration per 100,000 population for Leon and 12 control municipalities.

13) Appendix Figure 6: Insurance Claims Synthetic Control Results

14) Appendix Figure 7. Synthetic Control Results for Vital Registration Deaths

15) Appendix Table 5. Interrupted Time Series Results

16) Appendix Figure 8. Interrupted Time Series Results for Police-Reported Collisions

17) Appendix Figure 9. Seasonally adjusted Interrupted Time Series Results of police-reported collisions

18) Appendix Table 6. Synthetic Control Results Average Differences

19) Appendix Figure 10. Controlled Interrupted Time Series Results of Police-Reported Collisions.

Quistberg DA, *et al. Inj Prev* 2024;0:1–8. doi: 10.1136/ip-2023-045019

Appendix Figure 1. Leon intervention and evaluation timeline for main analyses.

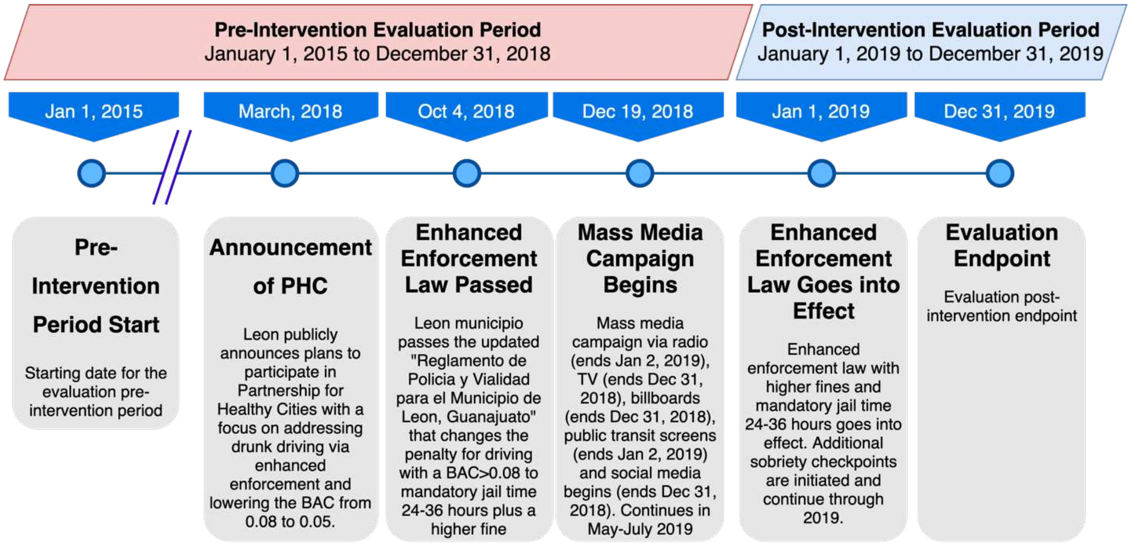

Appendix Table 1. Description of key predictors used to create synthetic Leon.

Predictor	Year	Description	Source
**Total urban area (hectares)**	2012	Total area within municipalities that is built-up, i.e., covered with human-built infrastructure and buildings	Global Urban Footprint (GUF) project, see:<br><br>Esch T, Heldens W, Hirner A, et al. Breaking new ground in mapping human settlements from space – The Global Urban Footprint. *ISPRS Journal of Photogrammetry and Remote Sensing* 2017;134:30-42. doi: https://doi.org/10.1016/j.isprsjprs.2017.10.012<br><br>Esch T, Schenk A, Ullmann T, et al. Characterization of Land Cover Types in TerraSAR-X Images by Combined Analysis of Speckle Statistics and Intensity Information. *IEEE Transactions on Geoscience and Remote Sensing* 2011;49(6):1911-25. doi: 10.1109/TGRS.2010.2091644<br><br>This variable is calculated based on 30m x 30m grid cells using the FRAGSTATS 4.2 software package:<br><br>FRAGSTATS v4: Spatial Pattern Analysis Program for Categorical and Continuous Maps. [program]. 4 version. Amherst, MA: University of Massachusetts, Amherst, 2012.
**Patch density (patches/100 hectares)**	2012	Number of urban patches (i.e., continuous built-up areas) within municipalities. Higher values indicate more urban fragmentation within a municipality	GUF and calculated using FRAGSTATS
**Intersection density (intersections/km^2)**	2018	Intersections were defined as nodes where 3 or more street segments met. Density is the number of intersections per geographic area within the municipality in kilometers squared	OpenStreetMap street network geographic files 2018.<br><br>Boeing G. OSMnx: New methods for acquiring, constructing, analyzing, and visualizing complex street networks. *Computers, Environment and Urban Systems* 2017;65:126-39. doi: https://doi.org/10.1016/j.compenvurbsys.2017.05.004
**Population density (persons/km^2)**	2010	Total population per geographic area within the municipality in kilometers squared	Consejo Nacional de Poblacion (CONAPO) population projections
**Alcohol outlets density (outlets/km^2)**	2016 and 2018	Total number of businesses selling alcohol per geographic area	National Statistics Directory of Economic Units (DENUE)
**Proportion of population male**	2018	Total population in living in the municipality that was male gender	CONAPO
**Proportion of population 15-34 years old**	2018	Total population in living in the municipality that was age 15 years to 34 years old.	CONAPO

Appendix Figure 2. Conceptual model of pre-intervention predictors, policy and road traffic collisions and deaths.

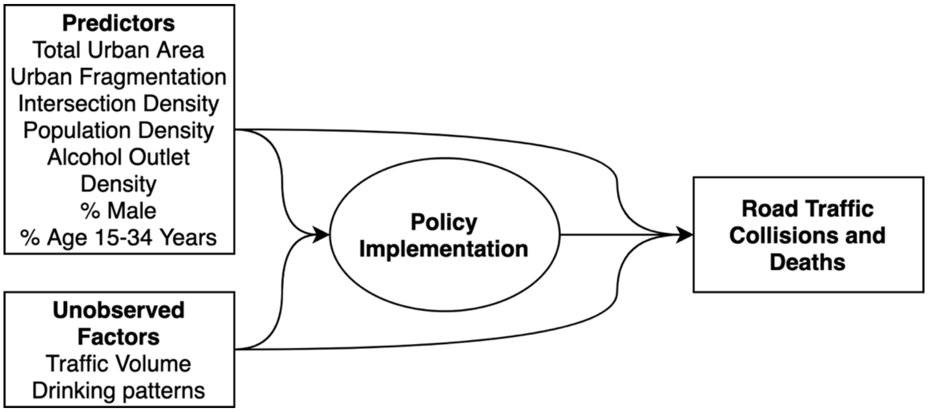

Appendix Table 2. Weight assigned to donor municipalities contributing to Synthetic Leon by outcome*.

		AXA Insurance Claims		Police-Reported Collisions				Vital Registration
Municipality	State	Total	Injuries	Total	Injuries	Fatal	Alcohol	Deaths
Acapulco	Guerrero	0.096	0.096	0	0.012	0.25	0.021	0.004
Aguascalientes	Aguascalientes	0.316	0.507	0.229	0.432	0.278	0.414	0.171
Benito Juarez	Quintana Roo	0	0	0	0.004	0	0.004	0.516
Centro	Tabasco	0	0.313	0	0.001	0	0.005	0
Coatzacoalcos	Veracruz	0.166	0	0.182	0.235	0.041	0.246	0.219
Morelia	Michoacan	0.36	0	0.302	0.165	0.331	0.183	0
Ocotlan	Jalisco	0	0	0	0.004	0	0.013	0
Tapachula	Chiapas	0	0	0	0.002	0	0.007	0.003
Tehuacan	Puebla	0	0	0	0.002	0	0.004	0.068
Torreon	Coahuila	0	0.084	0.141	0.061	0	0.081	0.017
Uruapan	Michoacan	0	0	0	0.003	0	0.01	0
Zamora	Michoacan	0.061	0	0.146	0.078	0.101	0.013	0

* Shaded cells indicate municipalities that contribute >0 weight to the synthetic Leon. Higher values indicate more contribution to synthetic Leon. All weights sum to 1.000.

Insurance Claims Denominator Estimations

Motor vehicle registration was derived from the Instituto Nacional de Estadistica y Geografia (INEGI, Mexico's national statistical agency) which collects data from states and municipalities of the number of registered vehicles in circulation and reports total number of registered vehicles annually. For registered automobiles covered by AXA, we used the state-level proportion of vehicles covered by AXA to adjust the automobile registration counts for each municipality within that state. Annual values were interpolated to months between each end of year December value. Overall, in Mexico, 28.6% of registered vehicles were covered by automobile insurance in 2018, with 24.9% in the state of Guanajuato where Leon is located.

Description of specific ICD-10 codes used to identity road traffic deaths

We considered all deaths coded with codes V01-V89 to be road traffic deaths with the exception of these codes:

V81.2 – Occupant of railway train or railway vehicle injured in collision with or hit by rolling stock

V81.3 – Occupant of railway train or railway vehicle injured in collision with other object

V81.4 – Person injured while boarding or alighting from railway train or railway vehicle

V81.5 – Occupant of railway train or railway vehicle injured by fall in railway train or railway vehicle

V81.6 – Occupant of railway train or railway vehicle injured by fall from railway train or railway vehicle

V81.7 – Occupant of railway train or railway vehicle injured in derailment without antecedent collision

V81.8 – Occupant of railway train or railway vehicle injured in other specified railway accidents

V81.9 – Occupant of railway train or railway vehicle injured in unspecified railway accident

Note: Y85.0 – Sequelae of Motor-Vehicle Accidents deaths were counted as road traffic deaths.

Appendix Table 3. Ill-defined ICD-10 death codes redistributed.

			Causes of Death to Redistribute to				
			V01-V89, Y850	V90-V98	W00-X58, Y40-Y84, Y86, Y88	X60-Y02, Y04-Y09, Y35-Y36, Y870, Y871, Y890, Y891	Y03, X82
			Land Transport Injuries	Other Transport Injuries	Other Unintentional Injuries	Intentional Injuries	Assault by motor vehicle
Ill-defined Causes to be Redistributed	Y34	Unspecified event, undetermined intent	Yes	Yes	Yes	Yes	Yes
	Y872	Sequelae of events of undetermined intent	Yes	Yes	Yes	Yes	Yes
	Y899	Sequelae of unspecified external cause	Yes	Yes	Yes	Yes	Yes
	X59	Ill-defined unintentional injuries	Yes	Yes	Yes	N/A	N/A
	Y859	Sequelae of other and unspecified transport injuries	Yes	Yes	N/A	N/A	N/A
	V99	Ill-defined transportation injuries	Yes	Yes	N/A	N/A	N/A
	Y32	Crashing of motor vehicle, undetermined intent	Yes	N/A	N/A	N/A	Yes

Appendix Figure 3. Time trends by municipality for police-reported collisions 2015-2019 by total, injury-causing, fatal, and alcohol-involved collisions, 2015-2019*.

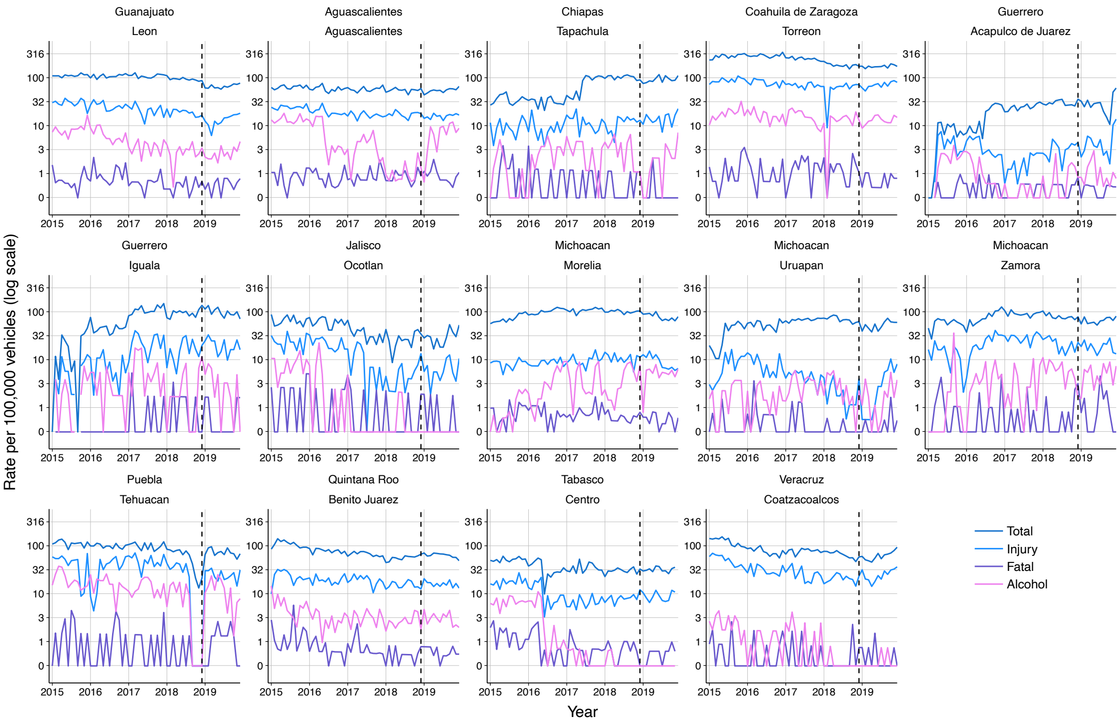

*** Y-axis is on the log scale.**

Description of Varying Time Periods for Sensitivity Analyses

As sensitivity to time of intervention analyses, we also examined 3 other scenarios for the pre-intervention/post-intervention periods: 1) setting the intervention date to October 1, 2018 when the enhanced enforcement law was passed (though not yet in effect) with the post-intervention evaluation period set to October 1, 2018 to December 31, 2019; 2) setting the intervention start date to December 1, 2018 (to reflect the mass media efforts in December 2018) with the post-intervention evaluation period as December 1, 2018 to December 31, 2019; and 3) removing October 1, 2018 to December 31, 2018 from the analysis and comparing the pre-intervention evaluation period as January 1, 2015 to September 30, 2018 to the post-intervention evaluation period as January 1, 2019 to December 31, 2019. These approaches allowed us to test the sensitivity of results to different specifications of the pre- and post-intervention period

Quistberg DA, *et al. Inj Prev* 2024;0:1–8. doi: 10.1136/ip-2023-045019

Appendix Table 4. Expanded version of Table 1 that includes Control Average results compared to synthetic Leon and Leon.

Predictor characteristics of Leon, Synthetic Control Units by outcome and average of donor control units. Values for predictors vary between Synthetic Control Units for each outcome due to differing weights of each donor municipality. See Appendix Table 1 for more details about each predictor and Appendix Table 4 for more details on the weights for each municipality within each Synthetic Control Unit

		Synthetic Leon (n=12)*							Control Average (n=12)
		AXA Insurance Claims		Police-Reported Collisions				Vital Registration Deaths	
Predictor	Leon	Total	Injuries	Total	Injuries	Fatal	Alcohol		
Intersection density, 2018**	19.2	15.6	10.9	15.8	13.2	16.2	16.3	11.0	9.9
Population density, 2018**	8,675	6,898	6,788	6,670	7,306	6,592	6,494	6,659	6933
Patch density, 2011 (fragmentation)	0.6	0.6	0.6	0.5	0.5	0.6	0.6	0.5	0.5
Alcohol outlets density, 2016/2018**	0.8	0.5	0.4	0.5	0.4	0.6	0.6	0.5	0.4
% population male, 2018	47.8	47.7	48.1	47.8	47.7	47.8	47.8	49.5	47.9
% population 15-34 years old, 2018	35.4	35.8	35.9	35.4	35.8	35.4	35.4	36.0	35.6
Total urban area, 2011 (KM2)	16,504	9,394	10,818	8,733	9,518	9,131	9,432	9,561	6925.4

*Because the synthetic control set weights may be different for different outcomes, the characteristics of each synthetic control vary and thus are shown by outcome.

**Per KM2

Quistberg DA, *et al. Inj Prev* 2024;0:1–8. doi: 10.1136/ip-2023-045019

Appendix Figure 4. Time trends by municipality for AXA insurance collisions claims 2015-2019 by total and injury-causing collisions*.

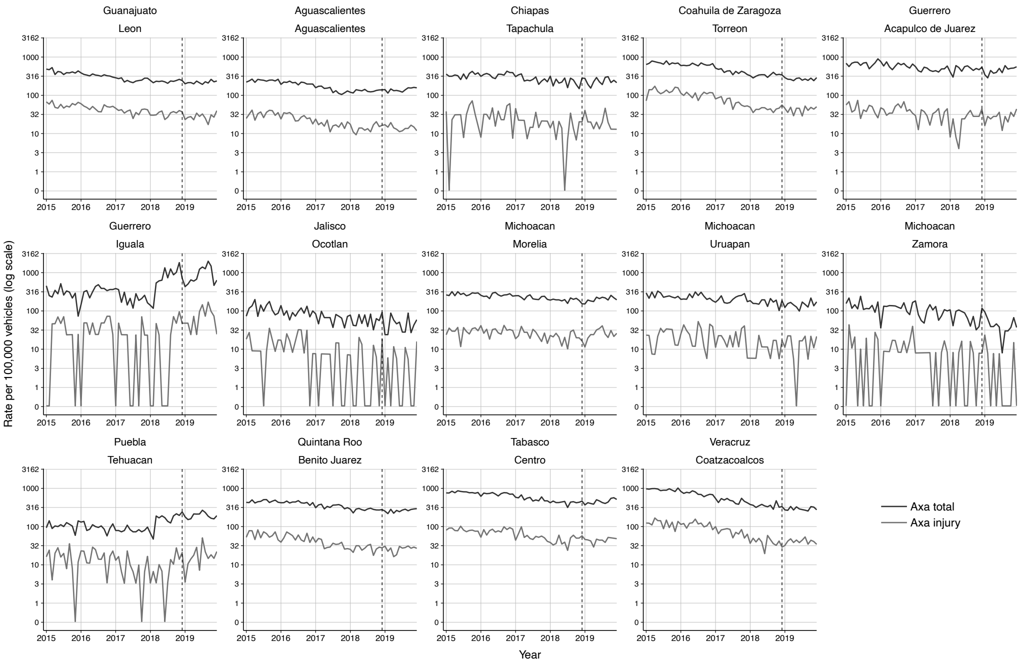

*** Y-axis is on the log scale.**

Appendix Figure 5. Bi-Monthly rates of road traffic deaths from vital registration per 100,000 population for Leon and 12 control municipalities.

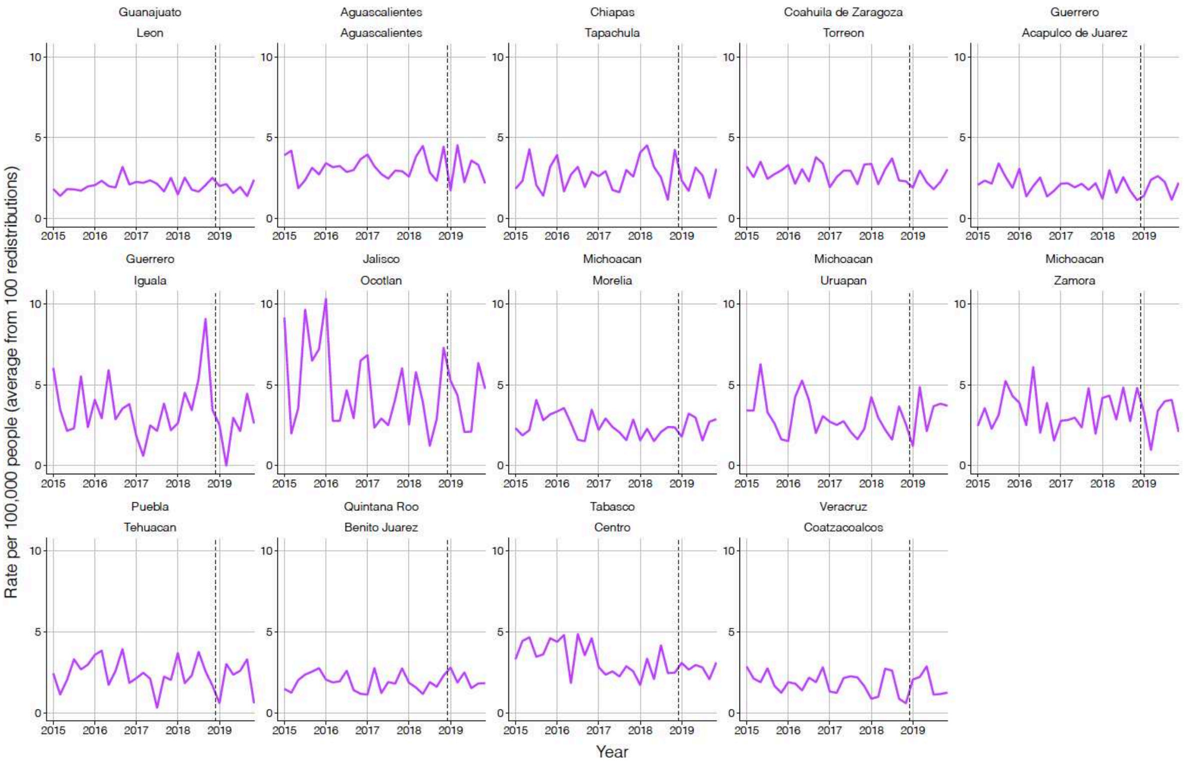

*** Y-axis is on the log scale.**

Appendix Figure 6: Insurance Claims Synthetic Control Results
Monthly rates of insurance claims collisions by a) total and b) injury in Leon vs *Synthetic Leon,* Feb 2015 to November 2019, before and after the implementation of an intervention to reduce drunk driving in January 2019. RMSPE = Root Mean Square Prediction Error, lower values indicate a closer match of synthetic Leon to Leon during the pre-intervention period. Placebo tests of insurance collision rates showing different (gap) by c) total and d) injury collisions by month between Leon and synthetic Leon and between each control municipality and its own synthetic control (placebo tests, labelled "Other Gaps"). Gap expressed as differences in rates per 100,000 insured vehicles. Note, all municipalities (12/12) had pre-intervention RMSPE<5 SD. Post/pre-RMSPE ratio distributions for e) Total collisions and f) Injury collisions.

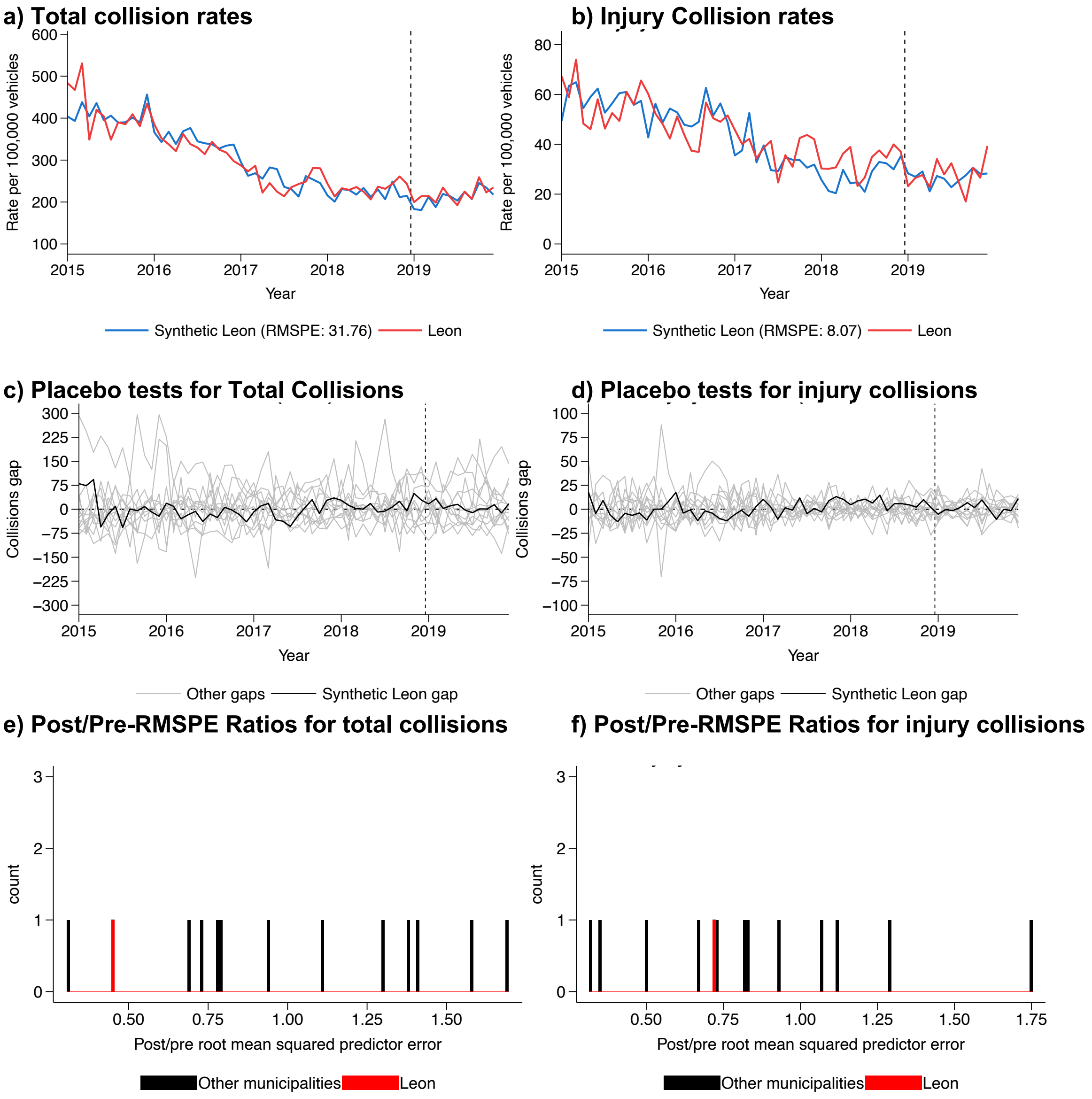

Appendix Figure 7. Post/Pre-RMSPE Distribution Plots for Police-Reported Collisions

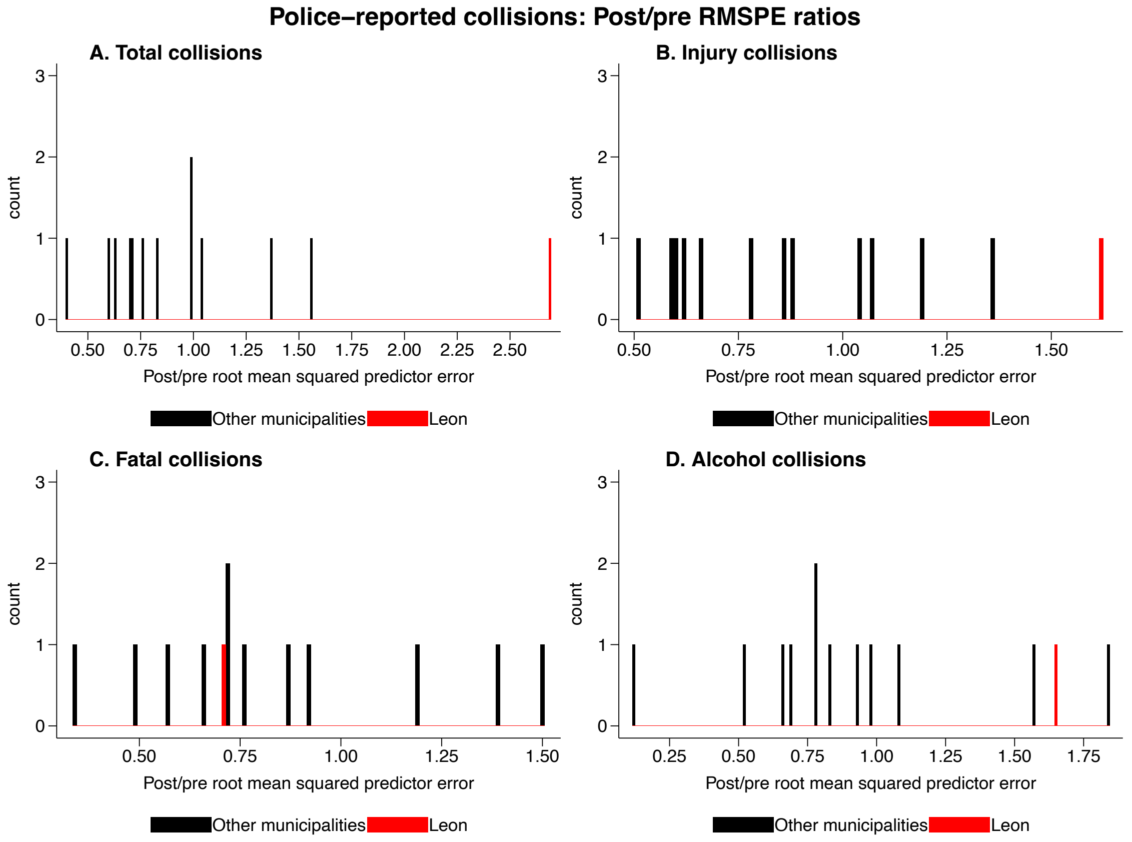

Appendix Figure 8. Synthetic Control Results for Vital Registration Deaths

A) Bi-Monthly rates of road traffic deaths from vital registration in Leon vs *Synthetic Leon,* Feb 2015 to November 2019, before and after the implementation of an intervention to reduce drunk driving in January 2019. RMSPE = Root Mean Square Prediction Error, lower values indicate a closer match of synthetic Leon to Leon during the pre-intervention period. B) Placebo tests of insurance collision rates showing different (gap between Leon and synthetic Leon and between each control municipality and its own synthetic control (placebo tests, labelled “Other Gaps”). Gap expressed as differences in rates per 100,000 insured vehicles. Note, 11/12 municipalities had pre-intervention RMSPE<5 SD. C) Post/pre RMSPE ratios.

a) Mortality rates

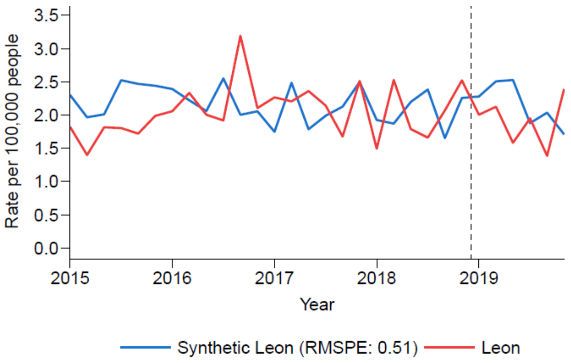

b) Placebo Tests

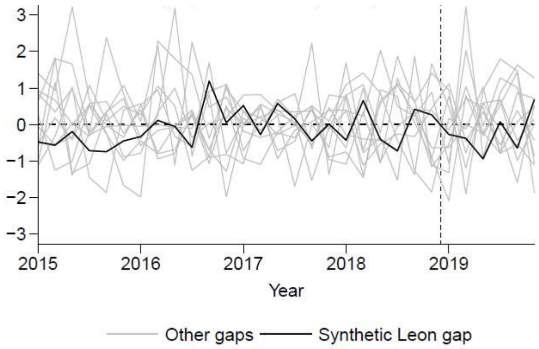

c) Post/pre-ratio RMPSE distribution plot

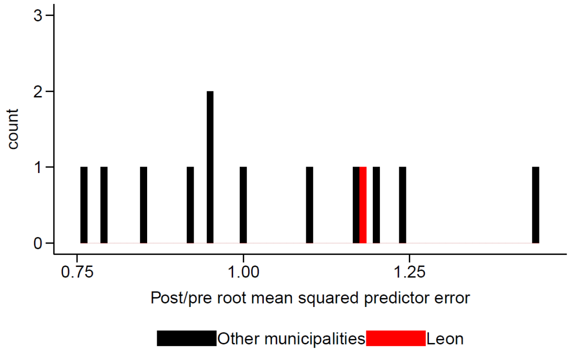

Quistberg DA, *et al. Inj Prev* 2024;0:1–8. doi: 10.1136/ip-2023-045019

Appendix Table 5. Interrupted Time Series Results
Incidence Rate Ratio (IRR) and 95% Confidence Intervals (95% CI) Estimates of each outcome obtained from GLM Poisson regression models in interrupted time series analysis of Leon.

	Unadjusted Models		Seasonally Adjusted Models	
	IRR (95% CI)	P value	IRR (95% CI)	P value
**POLICE REPORTED COLLISIONS**				
**Total Collisions**				
Intervention Present				
No	1.00		1.00	
Yes	0.62 (0.55,0.70)	<0.01	0.65 (0.57,0.73)	<0.01
Pre-Intervention Time Trend (per month)	1.00 (0.99,1.00)	<0.01	1.00 (0.99,1.00)	<0.01
Post-Intervention Time Trend (per month)	1.03 (1.01,1.04)	<0.01	1.02 (1.00,1.04)	0.02
**Injury Collisions**				
Intervention Present				
No	1.00		1.00	
Yes	0.56 (0.42,0.74)	0.03	0.56 (0.41,0.75)	<0.01
Pre-Intervention Time Trend (per month)	0.99 (0.98,0.99)	<0.01	0.99 (0.98,0.99)	<0.01
Post-Intervention Time Trend (per month)	1.08 (1.04,1.12)	0.03	1.09 (1.04,1.12)	<0.01
**Fatal Collisions**				
Intervention Present				
No	1.00		1.00	
Yes	0.46 (0.14, 1.45)	0.19	0.70 (0.23, 2.15)	0.53
Pre-Intervention Time Trend (per month)	0.98 (0.97, 0.99)	0.04	0.98 (0.97, 0.99)	0.01
Post-Intervention Time Trend (per month)	1.11 (0.96, 1.28)	0.17	1.05 (0.91, 1.21)	0.52
**Alcohol Collisions**				
Intervention Present				
No	1.00		1.00	
Yes	0.80 (0.45, 1.42)	0.45	0.95 (0.54, 1.66)	0.85
Pre-Intervention Time Trend (per month)	0.97 (0.96, 0.98)	<0.01	0.97 (0.96, 0.98)	<0.01
Post-Intervention Time Trend (per month)	1.09 (1.02 , 1.18)	0.02	1.07 (1.00, 1.15)	0.07
**VITAL REGISTRATION ROAD TRAFFIC DEATHS**				
Intervention Present				
No	1.00		1.00	
Yes	0.85 (0.62, 1.17)	0.325	0.885 (0.62, 1.27)	0.508
Pre-Intervention Time Trend (per month)	1.01 (0.99, 1.02)	0.246	1.01 (0.99, 1.02)	0.331
Post-Intervention Time Trend (per month)	1.00 (0.91, 1.09)	0.915	0.984 (0.89, 1.09)	0.755

Quistberg DA, *et al. Inj Prev* 2024;0:1–8. doi: 10.1136/ip-2023-045019

Appendix Figure 9. Interrupted Time Series Results for Police-Reported Collisions
Seasonally adjusted modelled of police-reported collisions (the dotted line after the intervention is the expected trend based on pre-intervention trends and red line is the observed trend).

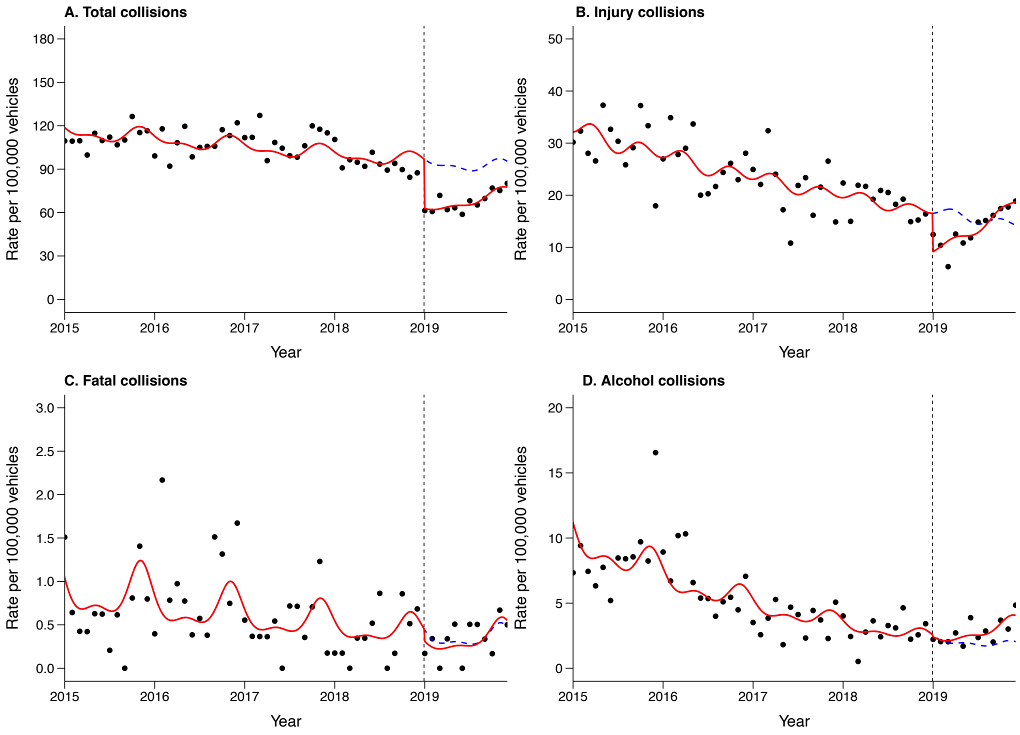

Quistberg DA, *et al. Inj Prev* 2024;0:1–8. doi: 10.1136/ip-2023-045019

Appendix Table 6. Synthetic Control Results Average Differences.
Average % difference between Leon and synthetic Leon in the post-intervention period in sensitivity analyses of different ways of defining the pre- and post-intervention periods

**Pre-Intervention Period**	**February 1, 2015 – September 30, 2018**	**February 1, 2015 – November 30, 2018**	**February 1, 2015 – December 18, 2018**
**Post-Intervention Period**	**October 1, 2018 – November 30, 2019**	**December 1, 2018 – November 30, 2019**	**December 19, 2019 – November 30, 2019**
**AXA Collision Claims**			
**Total**	+7.5%	+4.5%	+5.9%
**Injuries**	+15.0%	-0.6%	+6.5%
**Police-Reported Collisions**			
**Total**	-15.5%	-17.7%	-19.3%
**Injuries**	-33.2%	-33.8%	-35.6%
**Fatal**	-35.1%	-39.5%	-43.2%
**Alcohol**	-46.4%	-49.8%	-52.2%

Quistberg DA, *et al. Inj Prev* 2024;0:1–8. doi: 10.1136/ip-2023-045019

Appendix Figure 10. Controlled Interrupted Time Series trends of the average donor control of each outcome versus Leon, unadjusted and adjusted for seasonality.

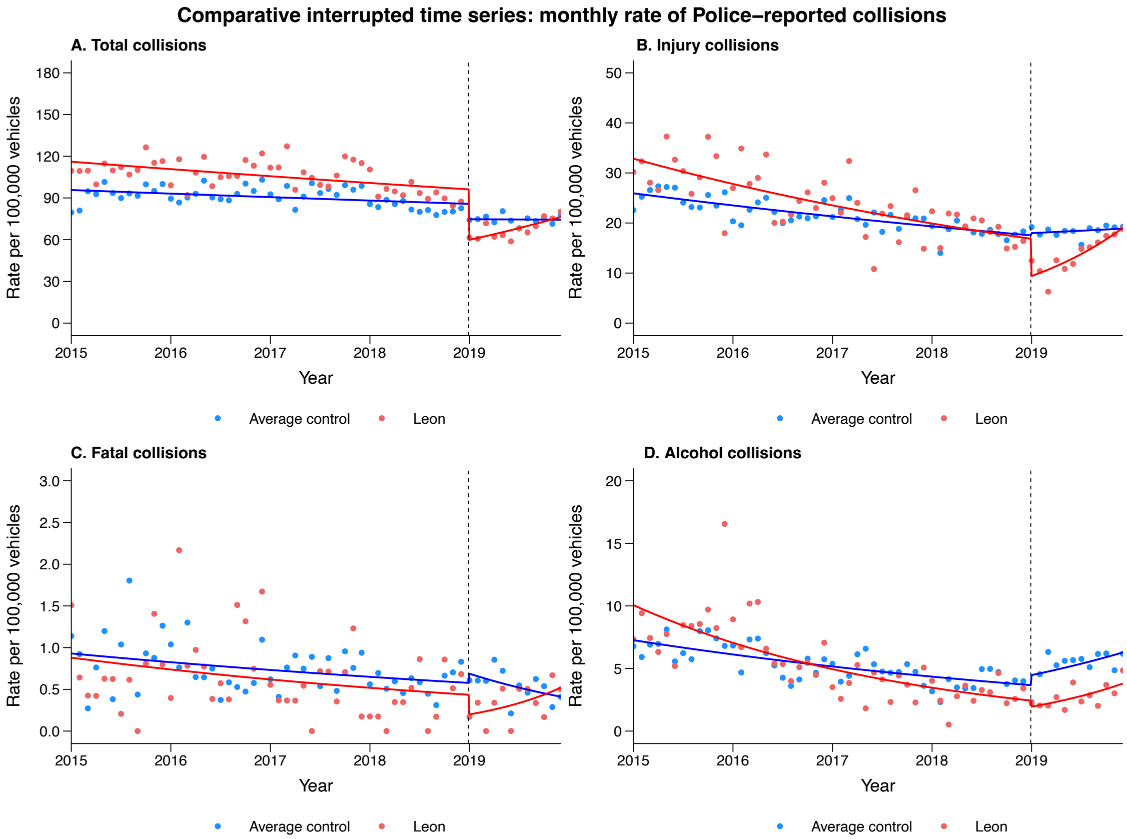

Quistberg DA, *et al. Inj Prev* 2024;0:1–8. doi: 10.1136/ip-2023-045019

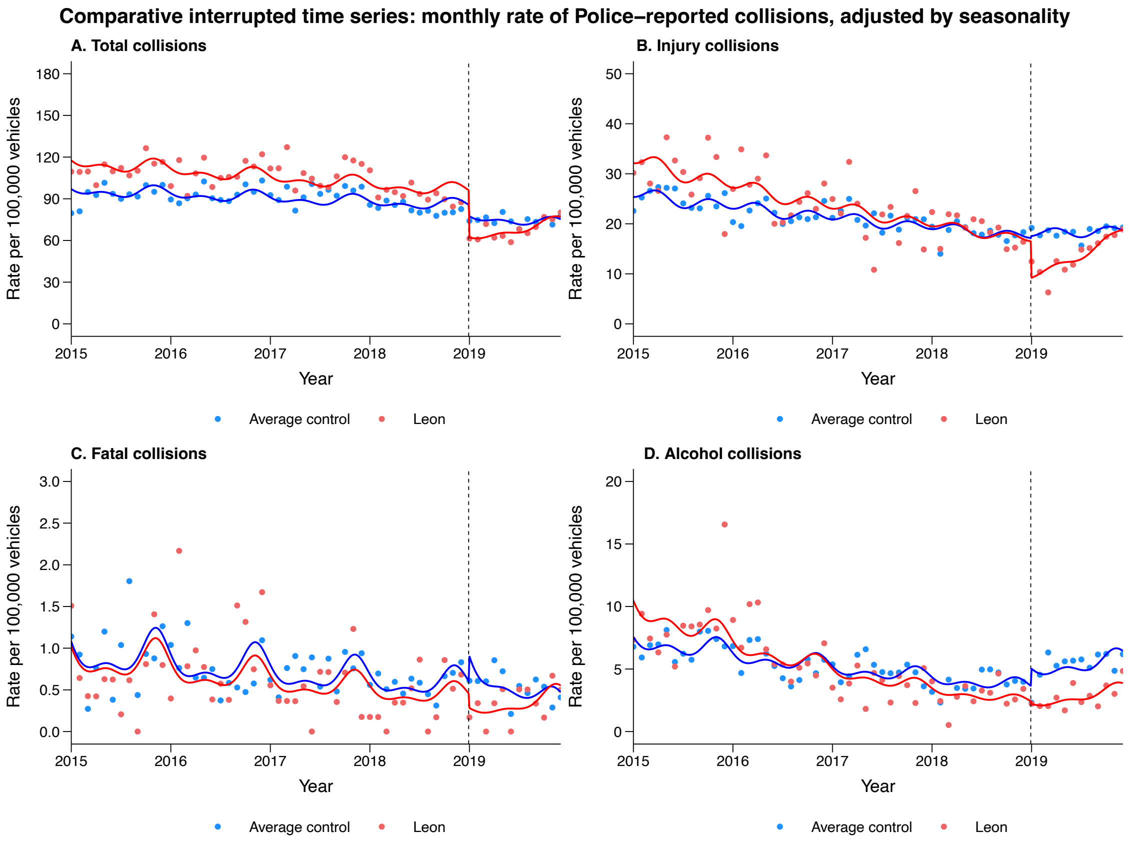

Quistberg DA, *et al. Inj Prev* 2024;0:1–8. doi: 10.1136/ip-2023-045019
